# Isolated Jejunal Crohn’s Disease Masquerading as Gastroparesis

**DOI:** 10.7759/cureus.26333

**Published:** 2022-06-25

**Authors:** Adalberto J Gonzalez, Sadaf Afraz, Jose Melendez-Rosado, Alison Schneider

**Affiliations:** 1 Gastroenterology, Cleveland Clinic Florida, Weston, USA; 2 Internal Medicine, Cleveland Clinic Florida, Weston, USA; 3 Gastroenterology and Hepatology, Eisenman & Eisenman Advanced, MD, Gastro Consultants, Lake Worth, USA

**Keywords:** inflammatory bowel disorder, small bowel obstruction, gastroparesis, isolated jejunal crohn’s disease, crohn’s disease

## Abstract

Isolated jejunal Crohn’s disease (IJCD) is a rare manifestation of small bowel inflammatory disease described in a few case reports. Due to challenges in diagnosis, this condition is overlooked or misdiagnosed in many instances. We present a case that was initially diagnosed as gastroparesis due to a gastric emptying study (GES) revealing delayed stomach clearance, with additional normal imaging and endoscopic examinations. After several imaging studies and a double-balloon enteroscopy (DBE), isolated Crohn’s disease was diagnosed and managed with surgical intervention. Isolated Crohn’s disease should be considered as a diagnosis in patients with gastroparesis to avoid delays in appropriate treatment and improve prognosis.

## Introduction

Crohn’s disease (CD) is a chronic inflammatory condition with variable clinical manifestation due to its ability to affect any part of the gastrointestinal (GI) tract [1]. Isolated jejunal Crohn’s disease (IJCD) is a rare small bowel manifestation representing only approximately 1% of cases of CD [2]. The diagnosis of IJCD with stricture can be made through imaging and endoscopy [2]. However, in the absence of visual evidence of a jejunal abnormality, IJCD may be misdiagnosed [3]. This abstract has been published in The American Journal of Gastroenterology.

## Case presentation

**Figure 1:**
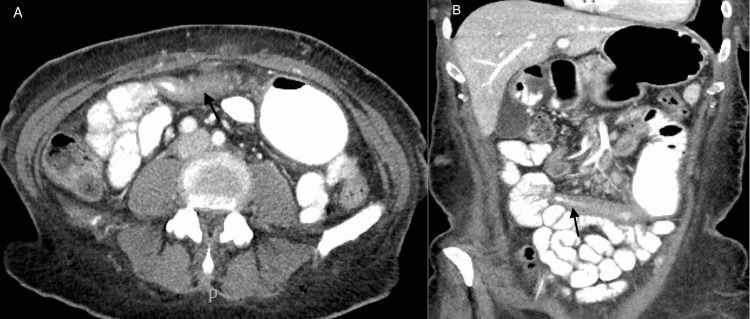
CT of the abdomen with oral contrast: (A) axial and (B) coronal images Concentric wall thickening of the proximal jejunum with stricture formation and proximal dilation.

**Figure 2:**
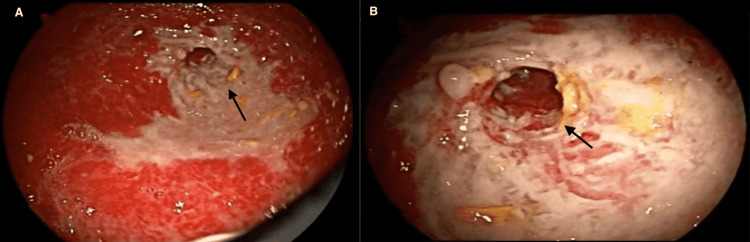
Endoscopic images of DBE A benign-appearing ulcerated stricture in the proximal jejunum. The intrinsic stricture was not transverse with the endoscope.

A 48-year-old female was referred to our gastroenterology clinic for a second opinion regarding gastroparesis. She initially presented 10 months prior to another gastroenterology clinic with complaints of postprandial epigastric abdominal pain, nausea, intermittent bilious emesis, early satiety, and constipation. Her past medical history was significant for gestational diabetes mellitus and hypothyroidism. She had no past surgical history and no significant family history. Her medications included levothyroxine and chlordiazepoxide-clidinium. Prior workup included a normal laboratory test including a chemistry panel, complete blood count, celiac serology, and inflammatory markers. The following were also normal: abdominal ultrasound with Doppler, computed tomography (CT) of the abdomen and pelvis, magnetic resonance enterography (MRE), esophagogastroduodenoscopy, and pancolonoscopy with ileoscopy. Further evaluation with a gastric emptying study (GES) showed 68% of residual food content in the stomach at four-hour intervals. She was diagnosed with idiopathic gastroparesis and managed with dietary changes, antiemetic therapy, a trial of metoclopramide as a prokinetic, and chlordiazepoxide-clidinium for abdominal pain. At our initial clinic visit, she reported ongoing nausea, vomiting, abdominal pain, and only being able to tolerate oral liquids but denied diarrhea or bloody stools. She had lost 165 pounds in the past 10 months. Physical examination showed normal vital signs, a BMI of 24 kg/m^2^, temporomandibular muscular wasting, and a soft, non-distended abdomen with tenderness to palpation in the upper abdominal area. She was started on domperidone with little success. Repeat laboratory tests revealed albumin of 2.9 g/dL, and a repeat upper endoscopy showed esophagitis, but it was otherwise normal. She was recommended to have repeat imaging with MRE; however, before completion, she presented to the emergency department with symptoms of generalized abdominal pain and emesis. A CT scan of the abdomen showed thickening of the proximal jejunum with stricture formation and proximal dilation, consistent with partial small bowel obstruction (Figure 1). A follow-up double-balloon enteroscopy (DBE) revealed erythema, linear ulcerations, and areas of stenosis in the proximal jejunum that could not be traversed by the endoscope (Figure 2). Multiple directed biopsies showed intestinal mucosa with ulceration, granulation tissue, and acute on chronic inflammation. The patient subsequently underwent a laparoscopic partial small bowel resection of the affected jejunal segment. Surgical pathology with similar findings of extensive ulceration, transmural inflammation, fibrinous serositis, and acute on chronic inflammation. The patient was diagnosed with IJCD. Her symptoms completely resolved after the small bowel resection. Repeat MRE at six-month follow-up showed no evidence of small bowel inflammation.

## Discussion

CD is an inflammatory bowel disease characterized by chronic transmural inflammation that may involve any part of the digestive tract, mostly found in the terminal ileum and proximal colon. Isolated jejunal disease is a rare clinical manifestation that represents approximately 1% of CD cases [2]. Early diagnosis may be challenging as it presents with nonspecific GI symptoms not initially appreciated on endoscopy or imaging [3]. We present a challenging case of isolated jejunal stricture in CD that was initially misdiagnosed as idiopathic gastroparesis. There are a few case reports on IJCD in the literature, but no reports specifically on IJCD mimicking symptoms of gastroparesis [2,3]. It is important to realize that delayed gastric emptying may be caused by a variety of mechanical and nonmechanical causes presenting with similar symptoms of nausea, vomiting, postprandial fullness, early satiety, abdominal distension, and abdominal pain located in the epigastrium [4]. One main reason for the initial misdiagnosis, in this case, was the fact that the clinical presentation of delayed gastric emptying related to idiopathic gastroparesis and IJCD appears very similar. The most common symptoms in IJCD are epigastric abdominal pain (82%), followed by diarrhea (52%), weight loss (25%), nausea (21%), and malaise (21%) [2]. Our patient also denied diarrhea, one of the most common symptoms in CD [5]. A lack of diarrhea in CD can often confound the diagnosis, as in this case. Another major diagnostic challenge was the lack of radiological and endoscopic evidence early in the presentation. In this case, both upper endoscopy and MRE were performed and were normal. MRE has a sensitivity of 75%-100% and a specificity of 91%-100% in diagnosing strictures in CD, but there is a suggestion that jejunal lesions are not as easily visualized on CT or MRI [5,6]. Colonoscopy was also performed and did not show any ileocolonic inflammation or fibrosis. CD in the absence of ileocolonic involvement is a rarely suspected diagnosis, and this likely also confounded the initial diagnosis. Lastly, our patient had markedly delayed gastric emptying on GES, which served as an anchoring bias to the initial diagnosis of idiopathic gastroparesis. In our patient, DBE was useful in clarifying the diagnosis of IJCD once it was seen on repeat CT imaging [7]. This is one of the additional imaging modalities that can be used to help clinch the diagnosis of IJCD. Wireless capsule endoscopy is another very sensitive test for finding abnormal mucosa but has low specificity for diagnosing CD and has the risk of becoming retained or impacted in cases of stricturing CD [8,9]. A 2008 prospective, blinded trial showed that the combination of different imaging modalities improved the sensitivity of diagnosing small bowel CD [10].

## Conclusions

In conclusion, our case highlights the importance of excluding small bowel CD in all patients with refractory gastroparesis, even in cases of unremarkable endoscopy and imaging. Combining and repeating multiple imaging and endoscopic modalities may be necessary to adequately diagnose small bowel CD without ileocolonic involvement, such as in IJCD. It is very important to look for mechanical causes such as IJCD as management, complications, and the overall prognosis are very different from idiopathic gastroparesis.
